# Supplementary material for: Multifunctional metal-organic framework-based nanoreactor for starvation/oxidation improved indoleamine 2,3-dioxygenase-blockade tumor immunotherapy
Source: Nat Commun. 2022 May 16;13:2688. doi: 10.1038/s41467-022-30436-y (PMC9110376; doi:10.1038/s41467-022-30436-y)
Supplement: Supplementary file 1 — Supplementary Information [file 41467_2022_30436_MOESM1_ESM.pdf]

Supplementary Information

Multifunctional metal-organic framework-based nanoreactor for starvation/oxidation improved indoleamine 2,3-dioxygenase-blockade tumor immunotherapy

Dai et al.

Supplementary experimental details

Materials. branched PEI (Mw 2 kDa), N-hydroxysuccinimide (NHS), 3-mercaptopropionic acid (MPA), methoxy poly(ethylene glycol) (mPEG-OH, Mn 5000 g/mol), and 1-MT were purchased from Sigma-Aldrich (Beijing, China). Dichloromethane (DCM), 4-dimethylaminopyridine (DMAP), N,N-dimethylformamide (DMF), and 1-ethyl-3-(3-dimethyl amino propyl) carbodiimide hydrochloride (EDC·HCl) were bought from J&K Scientific Ltd. Mouse anti-Tubulin, mouse anti-Bcl-2, mouse anti-Cyt-C, secondary antibodies of horseradish peroxidase-conjugated goat anti-mouse IgG and goat anti-rabbit IgG, and mouse anti-Bax were obtained from Boster Biological Technology Co., Ltd (Wuhan, China). ActinRed™ 555 ReadyProbes™ Reagent was supplied by Thermo Fisher Scientific (Waltham, USA). Primary antibodies including anti-mTOR and mouse anti-STAT-3, rabbit anti-IDO, and anti-Ki-67 were obtained from Proteintech Group, Inc. (Wuhan, China) and Cell Signaling Technology, Inc. respectively.

Characterization. The structures and molecular weights of the multipolymers were measured by ^{1}H NMR (Bruker Avance 500 MHz, Swiss), mass spectrometry (Waters Acquity SQ Detector UPLC-MS), gel-permeation chromatography (GPC) and Fourier transforms infrared spectroscopy (FTIR, model 6300, BioRad Co. Ltd., USA). The morphology of multipolymers was characterized by transmission electron microscopy (TEM, LIBRA 200 CS, Carl Zeiss Co., Germany). The sizes and size distribution of multipolymers were measured by zeta potential measurement (Nano ZS90 Zeta sizer, Malvern Instruments Co. Ltd, UK) equipped with dynamic light scattering (DLS). Brunauer-Emmett-Teller (BET) measurements were performed by an ASAP2050 system.

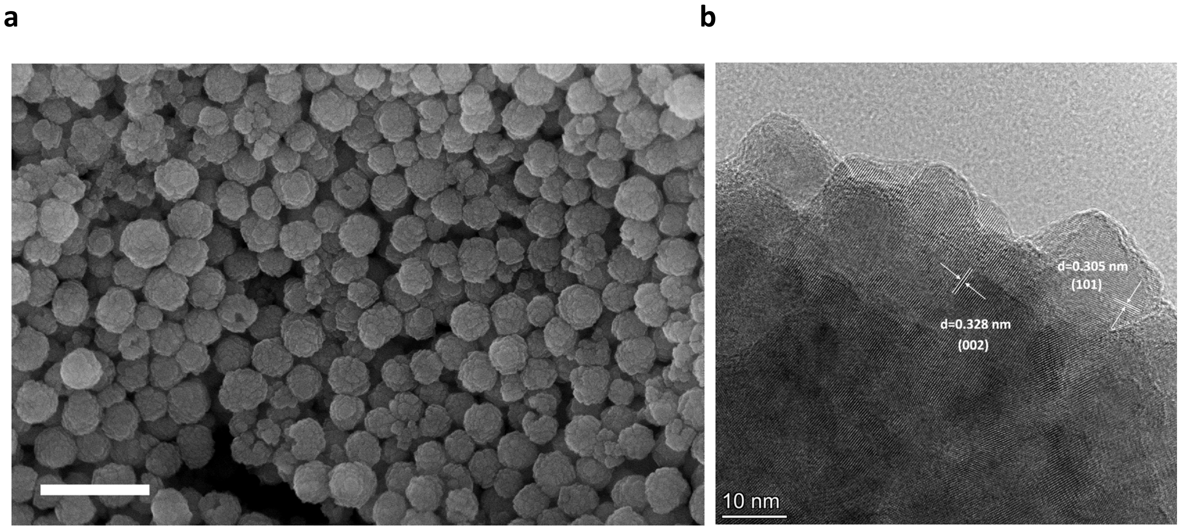

Supplementary Fig. 1. SEM and HRTEM images. (a) SEM and (b) HRTEM images of Mn-DTA. Scale bars: 200 nm for a, and 10 nm for b.

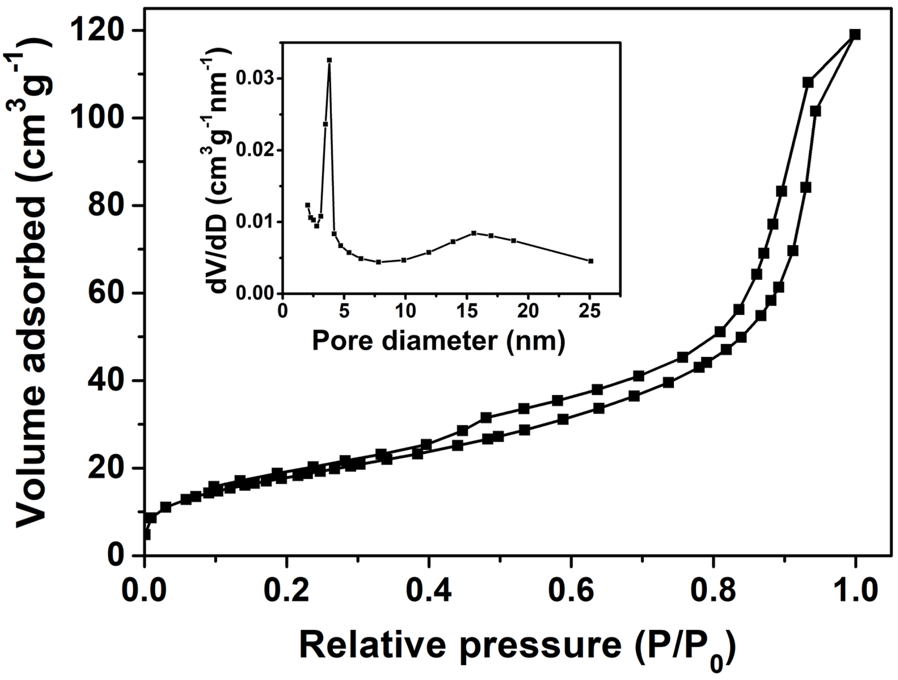

Supplementary Fig. 2. N_2 adsorption/desorption isotherm curves. N_2 adsorption/desorption isotherm curves of Mn-DTA.

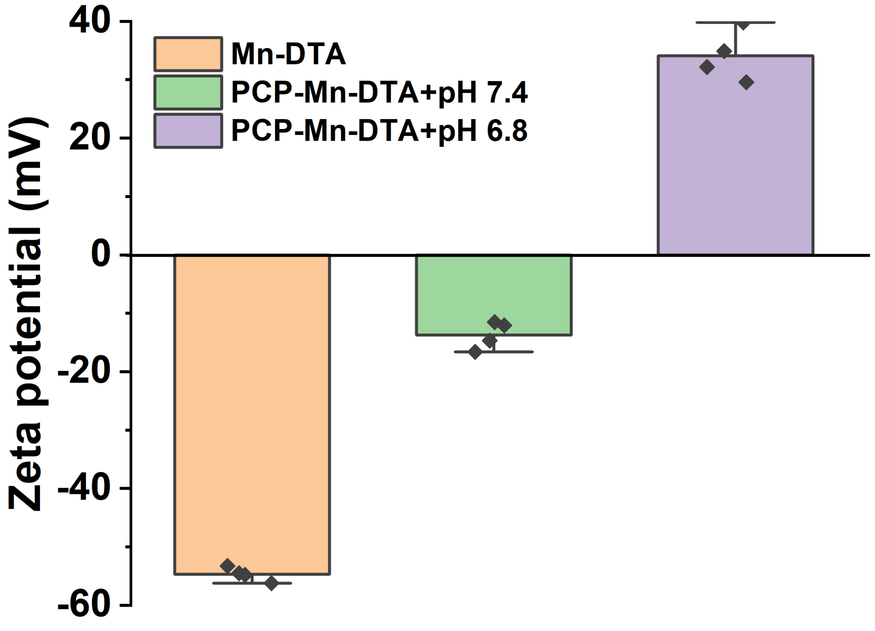

Supplementary Fig. 3. Zeta potential analysis. Zeta potential values of Mn-DTA and PCP-Mn-DTA nanoparticles at pH 7.4 and 6.8, respectively. Data represent mean ± SD (n = 4 independent samples).

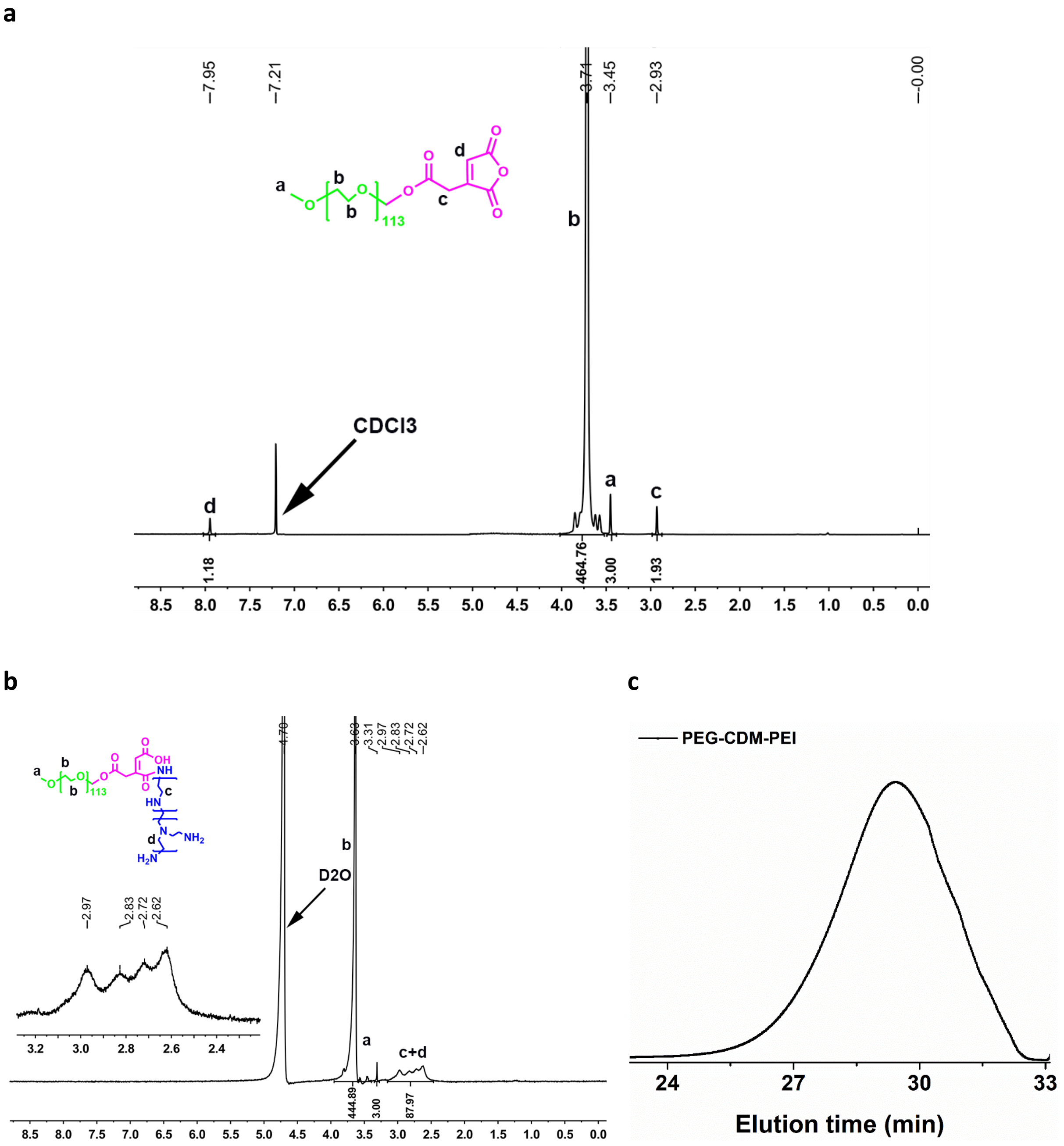

Supplementary Fig. 4. NMR and GPC studies. (a) ^{1}H NMR spectrum of PEG-CDM; (b) ^{1}H NMR spectrum and (c) GPC trace (THF as medium) of PEG-CDM-PEI copolymer.

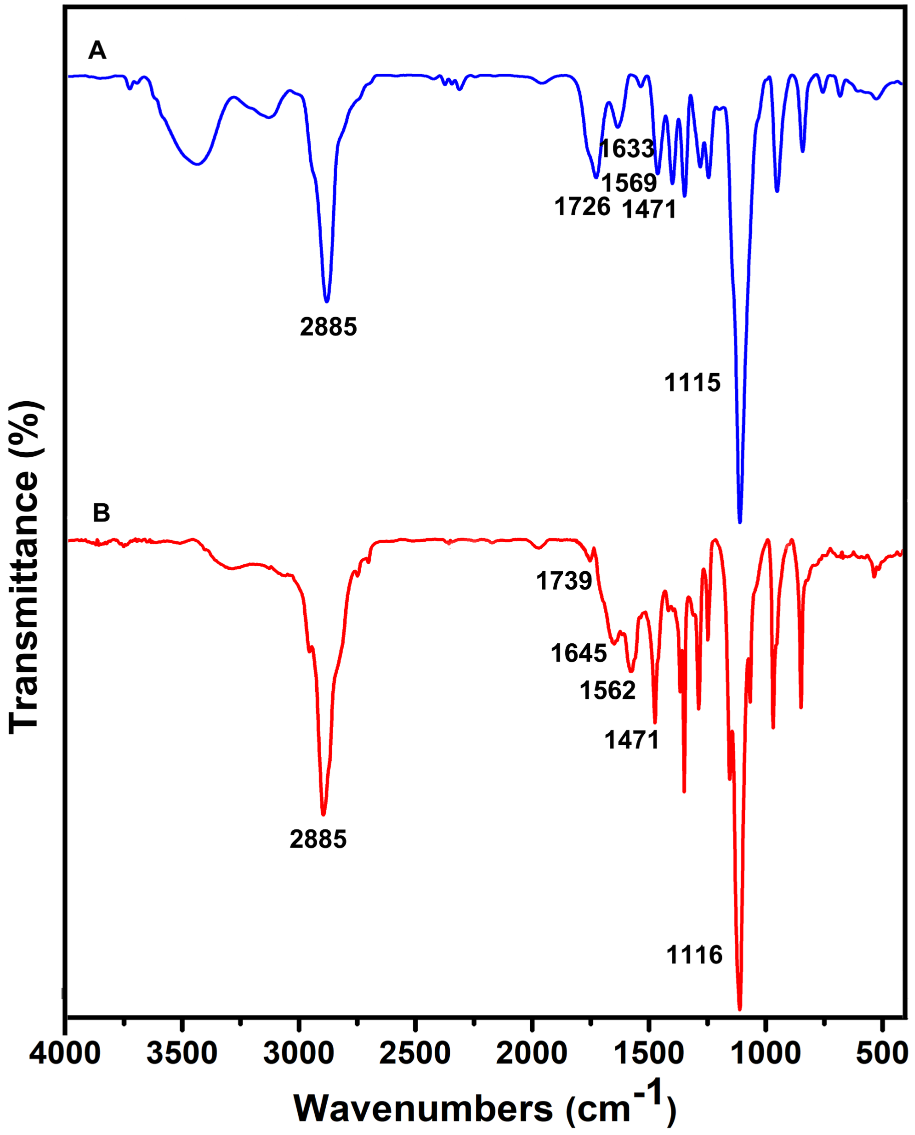

Supplementary Fig. 5. FTIR spectra. FTIR spectra of (a) PEG-CDM and (b) PEG-CDM-PEI copolymers.

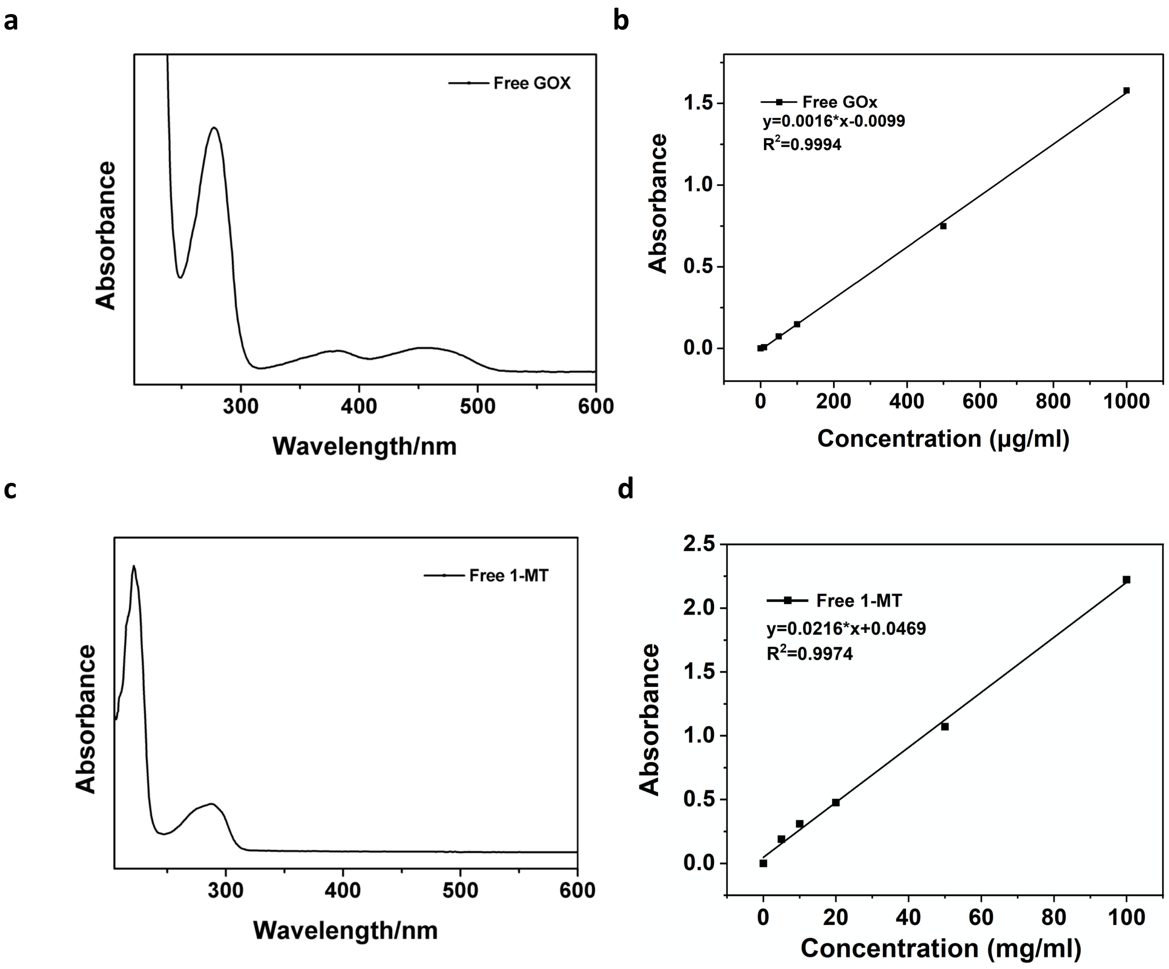

Supplementary Fig. 6. Absorbance spectra. Absorbance spectra and corresponding standard curves of (a,b) free GOx and (c,d) free 1-MT, respectively.

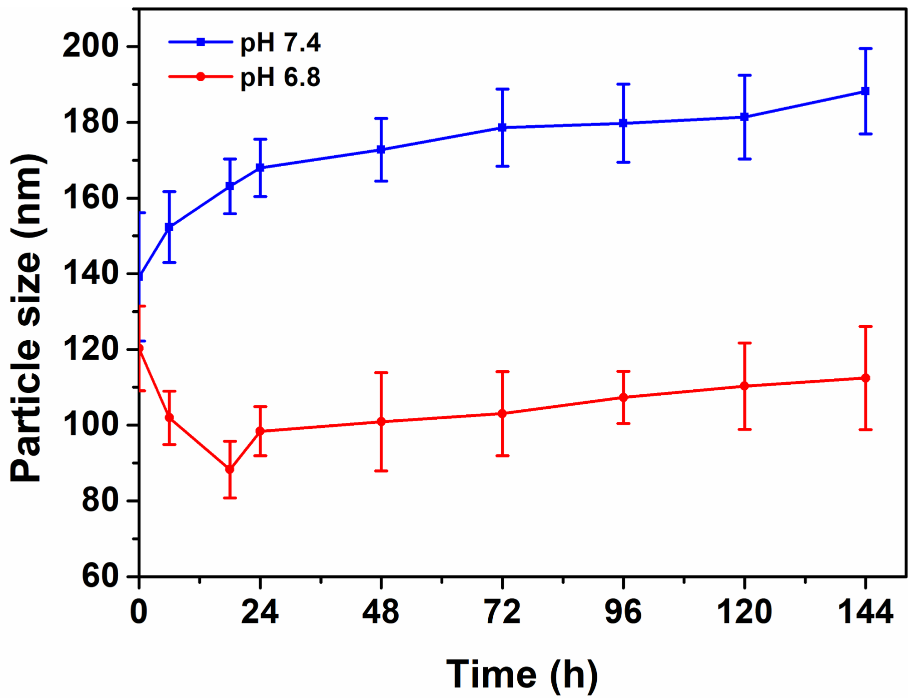

Supplementary Fig. 7. DLS analysis. Particle sizes of PCP-Mn-DTA nanosystem incubated in serum at pH 7.4 and 6.8 for 6 days, detected by DLS. Data represent mean ± SD (n = 4 independent samples).

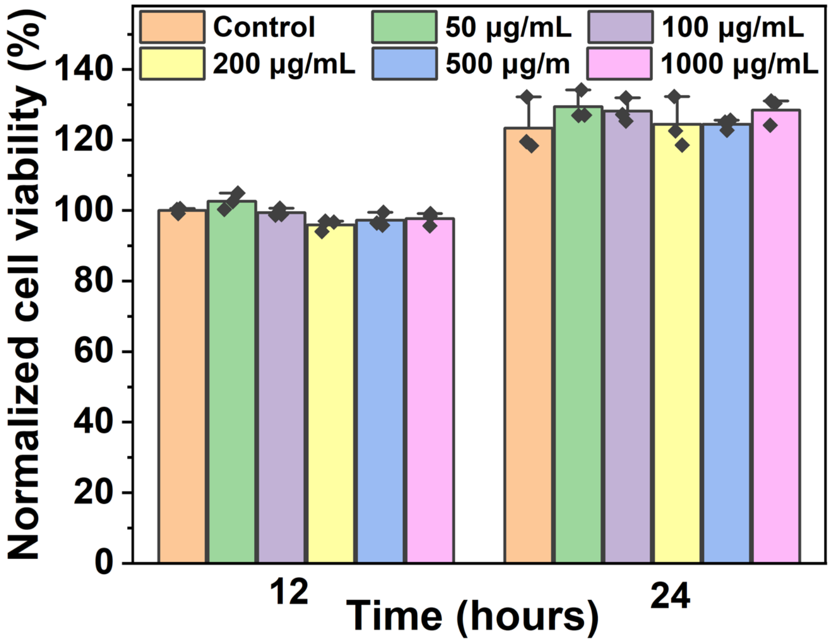

Supplementary Fig. 8. Cell viability study. Cell viability of B16F10 cells treated with PCP-Mn-DTA blank nanocarrier upon different dosages for 12 h and 24 h, respectively. Data represent mean ± SD (n = 3 biologically independent samples).

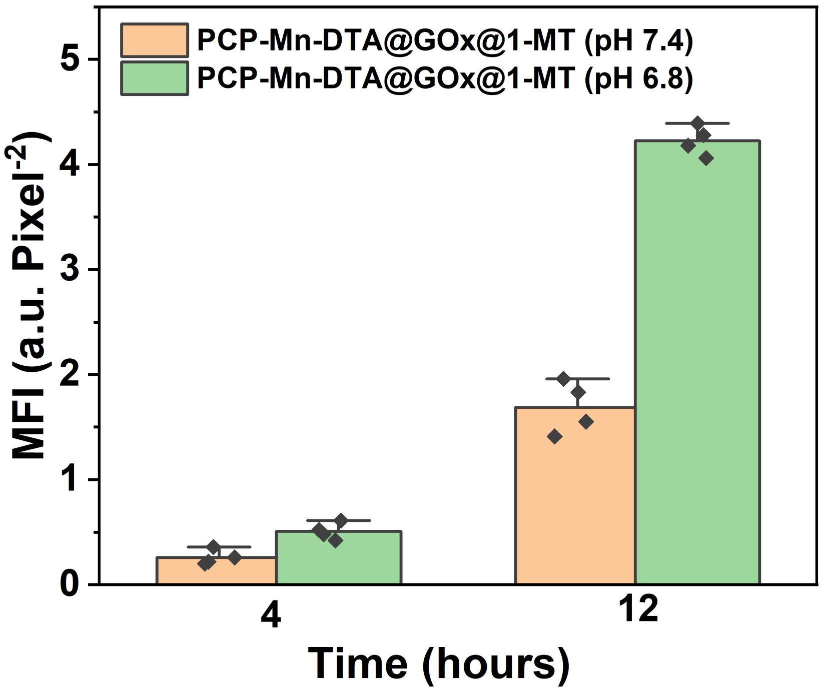

Supplementary Fig. 9. Quantitative fluorescence intensity analysis. Mean fluorescence intensity (MFI) of PCP-Mn-DTA@GOx@1-MT labelled with FITC in B16F10 MCSs after respective incubation at pH 7.4 or 6.8 for 4 h and 12 h. Data represent mean ± SD (n = 4 biologically independent samples).

a

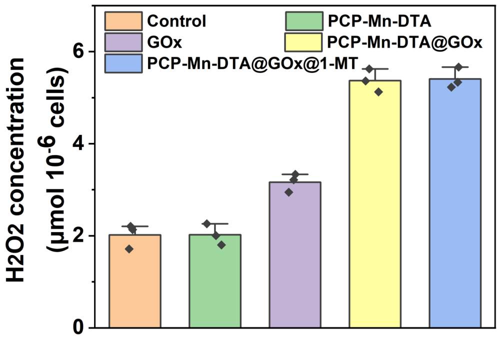

b

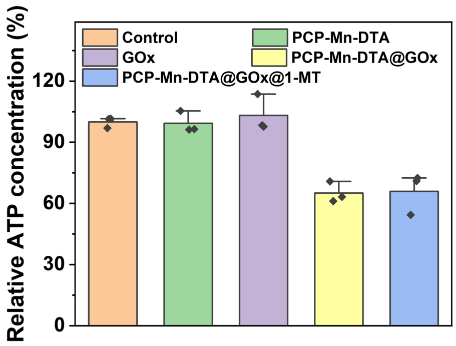

Supplementary Fig. 10. Concentrations of H_2O_2 and ATP. Concentrations of (a) H_2O_2 and (b) ATP in B16F10 cells after different treatments. Cells without any treatment were defined as a control. Data represent mean ± SD (n = 3 biologically independent samples).

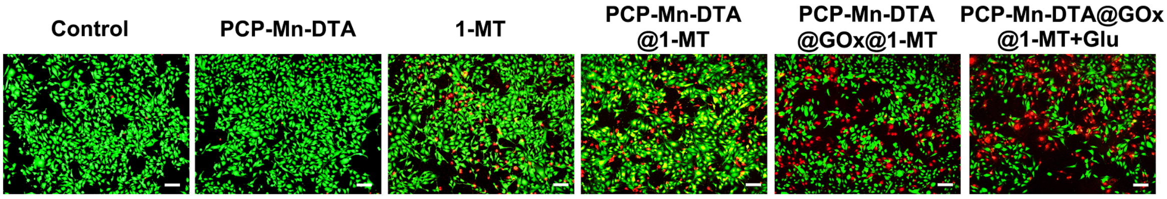

Supplementary Fig. 11. Live-dead imaging study. Live-dead assay of B16F10 tumor cells after difference treatments for 24 h, with images representative of 3 experiments. Glu: glucose. Scale bar: 100 µm.

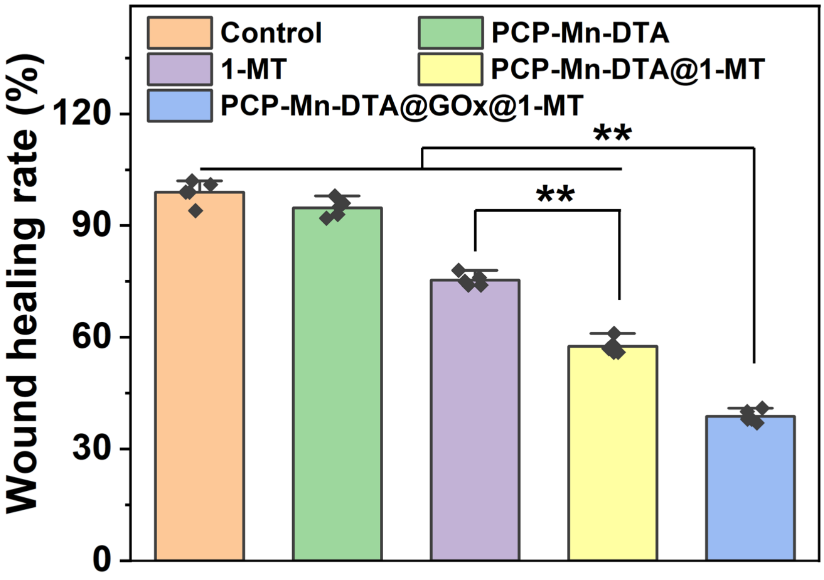

Supplementary Fig. 12. Quantitative wound healing analysis. Quantitative analysis on the wound healing assays of B16F10 cells treated with PCP-Mn-DTA, 1-MT, PCP-Mn-DTA@1-MT, and PCP-Mn-DTA@GOx@1-MT. Data represent mean ± SD (n = 6 biologically independent samples). P-values were determined by unpaired Student's t-test (two-tailed), $^{**}p < 0.01$.

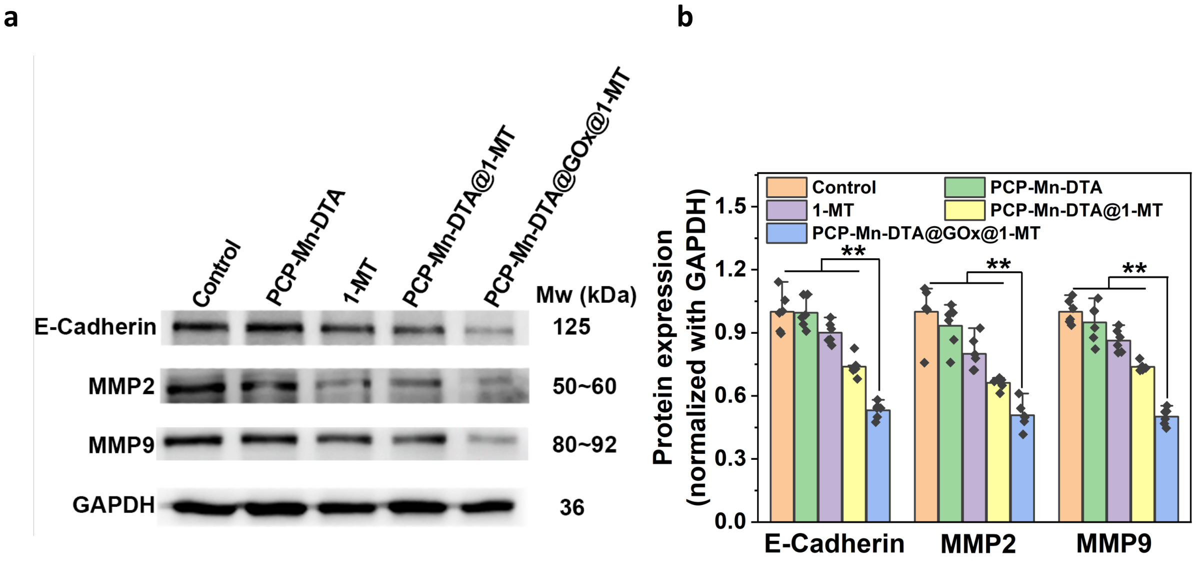

Supplementary Fig. 13. Tumor invasion-associated proteins. Tumor invasion-associated proteins (E-Cadherin, MMP2, MMP9) in B16F10 cells after the incubation with various treatments examined by (a) western blotting and (b) the related quantitative analysis. Data represent mean ± SD (n = 6 biologically independent samples). P-values were determined by unpaired Student's t-test (two-tailed), $^{**}p < 0.01$.

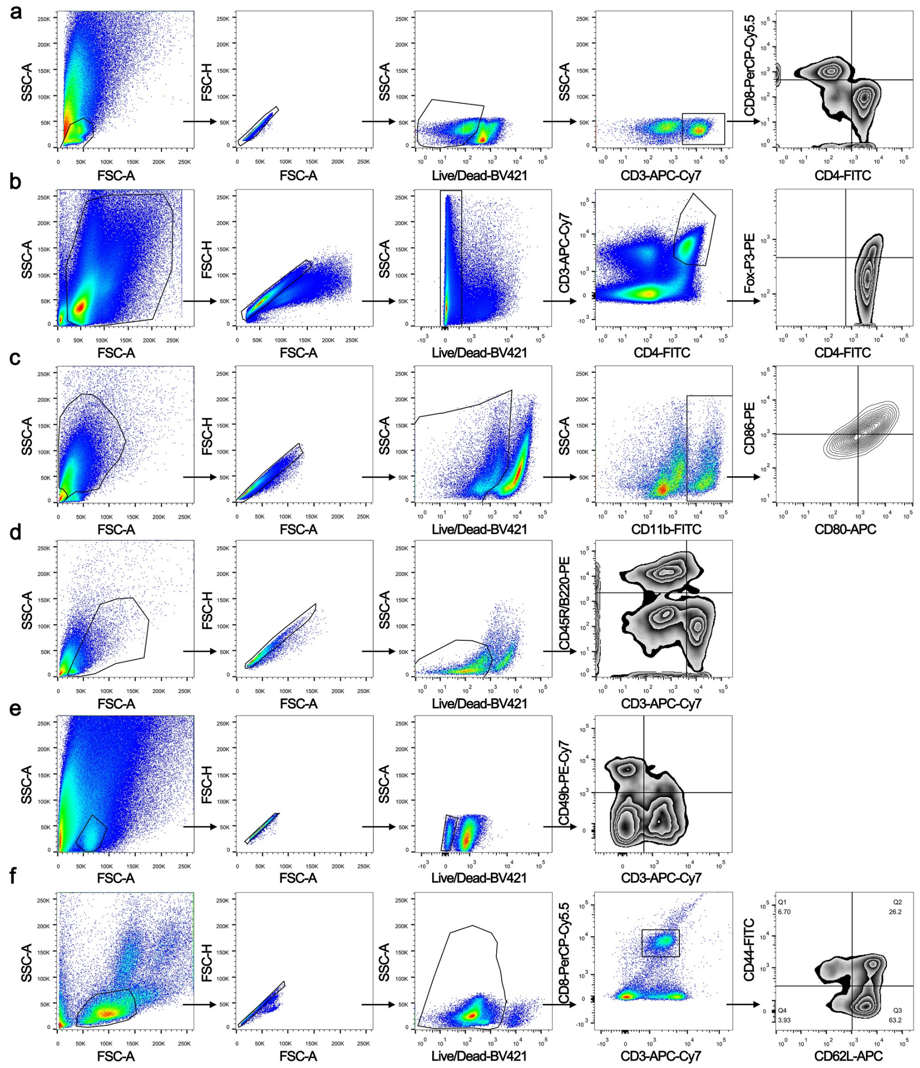

Supplementary Fig. 14. Gating strategy results. Gating strategies for assessing B16F10 and 4T1 tumor-infiltrating (a) CD8⁺/CD4⁺ T cells, (b) Treg cells, (c) dendritic cells, (d) B cells, (e) NK cells, and (f) central and effector memory cells by following the indicated treatments.

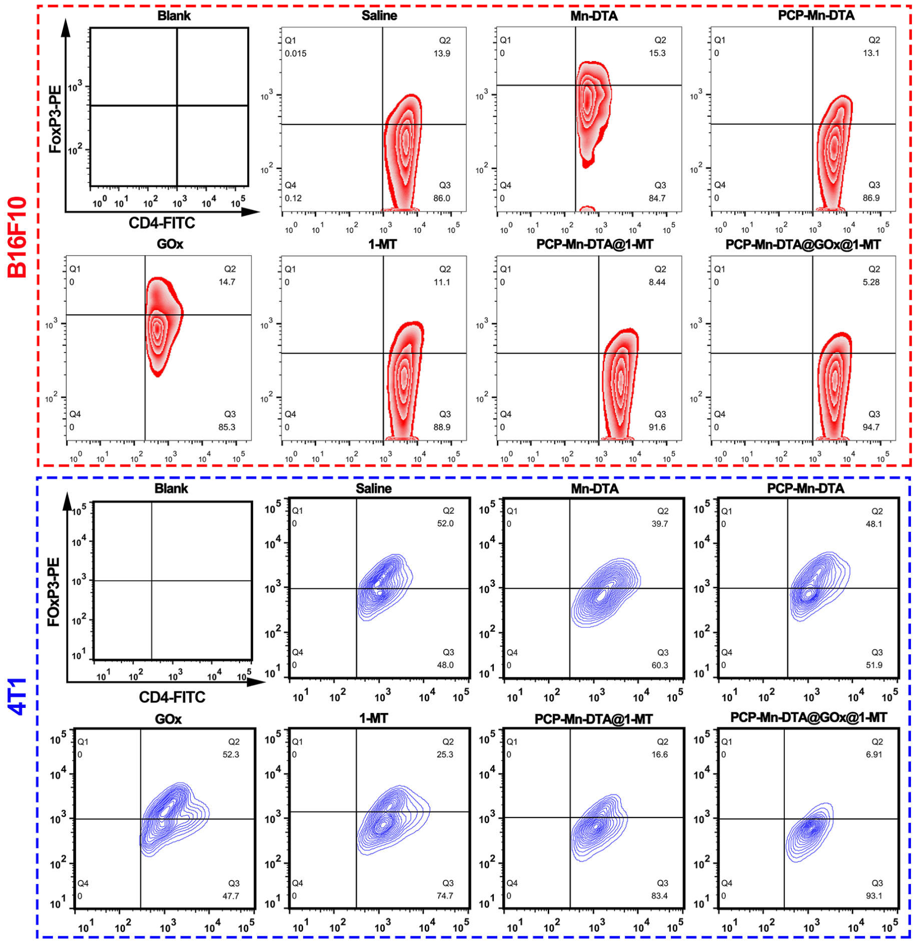

Supplementary Fig. 15. Quantitative FCM analysis. FCM examination of tumor-infiltrating Treg cells ($CD3^+CD4^+FoxP3^+$) in the B16F10 and 4T1 tumor-bearing mice after 4 d post-administration, respectively.

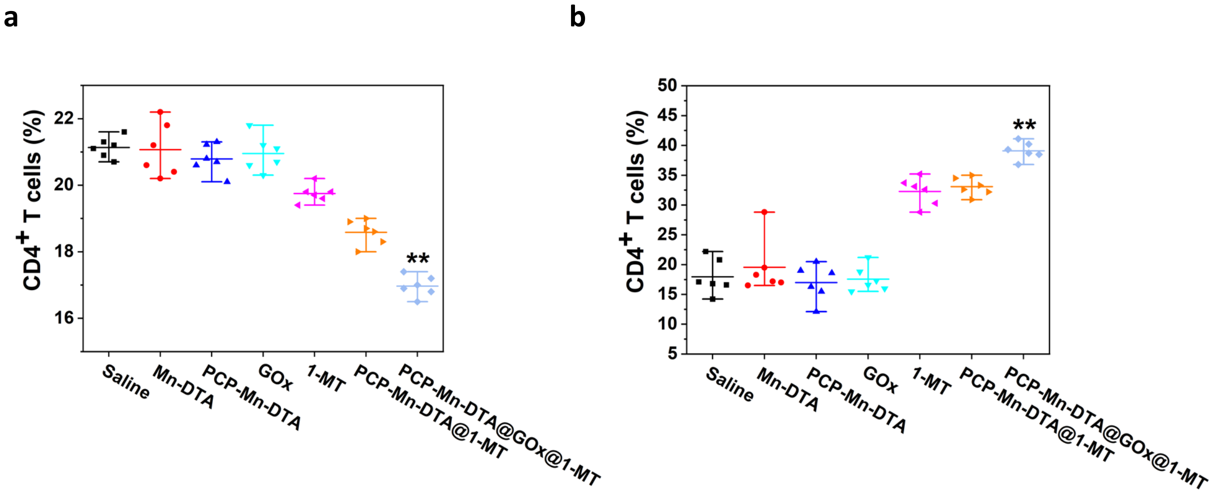

Supplementary Fig. 16. Quantitative FCM analysis. Quantitative FCM analysis of tumor-infiltrating $CD4^+$ T cells in (a) B16F10 and (b) 4T1 tumor-bearing mice after 4 d post-administration, respectively. Data represent mean ± SD (n = 6 biologically independent samples). P-values were determined by unpaired Student's t-test (two-tailed), **$p < 0.01$.

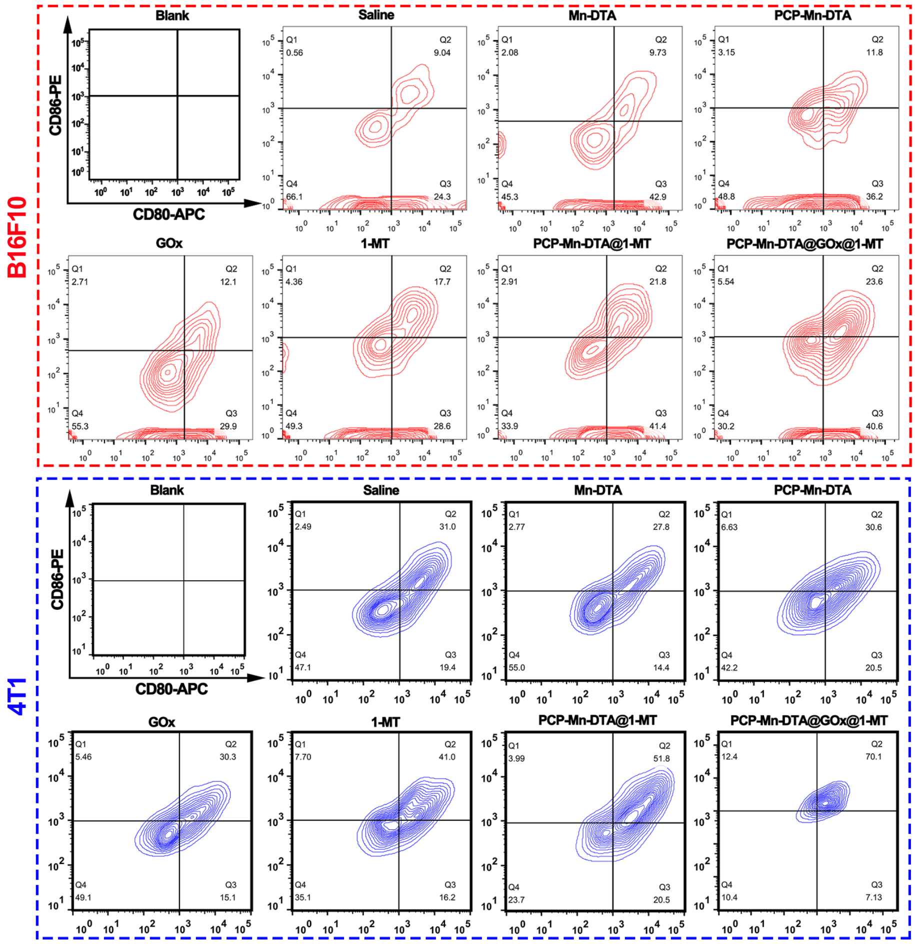

Supplementary Fig. 17. Quantitative FCM analysis. Quantitative FCM analysis of tumor-infiltrating DC cells ($CD11b^+CD80^+CD86^+$) in B16F10 and 4T1 tumor-bearing mice after 4 d post-administration, respectively.

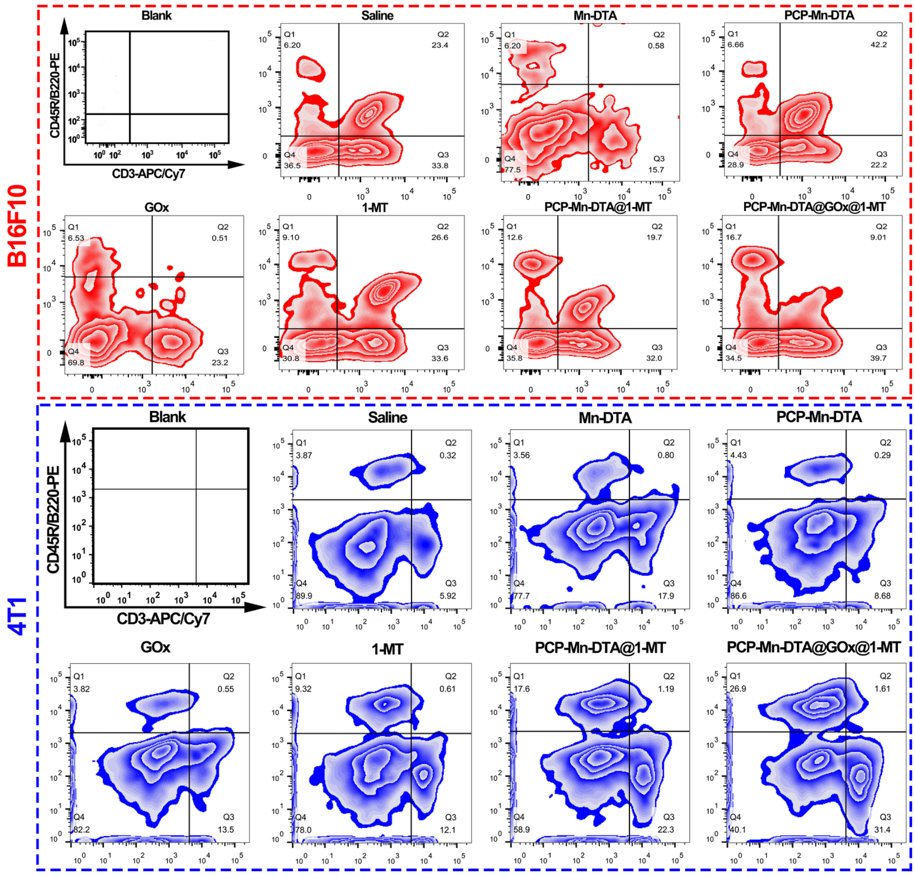

Supplementary Fig. 18. Quantitative FCM analysis. FCM examination of tumor-infiltrating B cells ($CD3^{-}CD45R/B220^{+}$) in B16F10 and 4T1 tumor-bearing mice after 4 d post-administration, respectively.

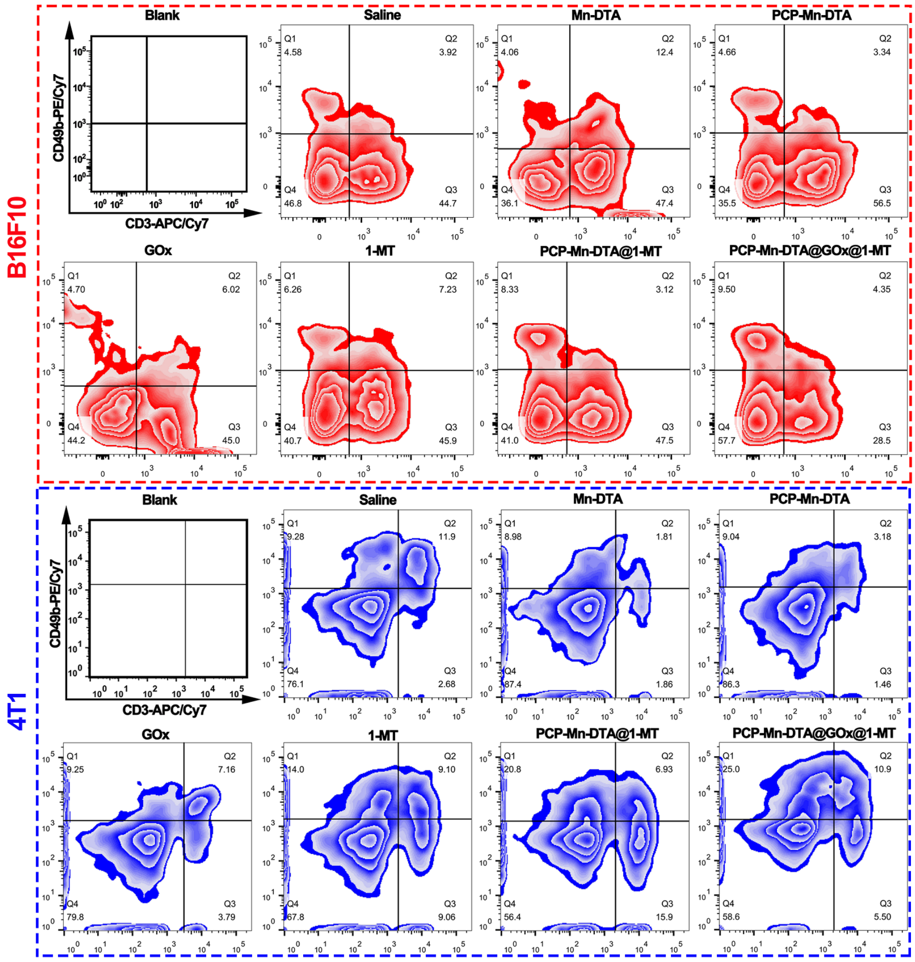

Supplementary Fig. 19. Quantitative FCM analysis. FCM examination of tumor-infiltrating NK cells ($CD3^{-}CD49b^{+}$) in B16F10 and 4T1 tumor-bearing mice after 4 d post-administration, respectively.

a b

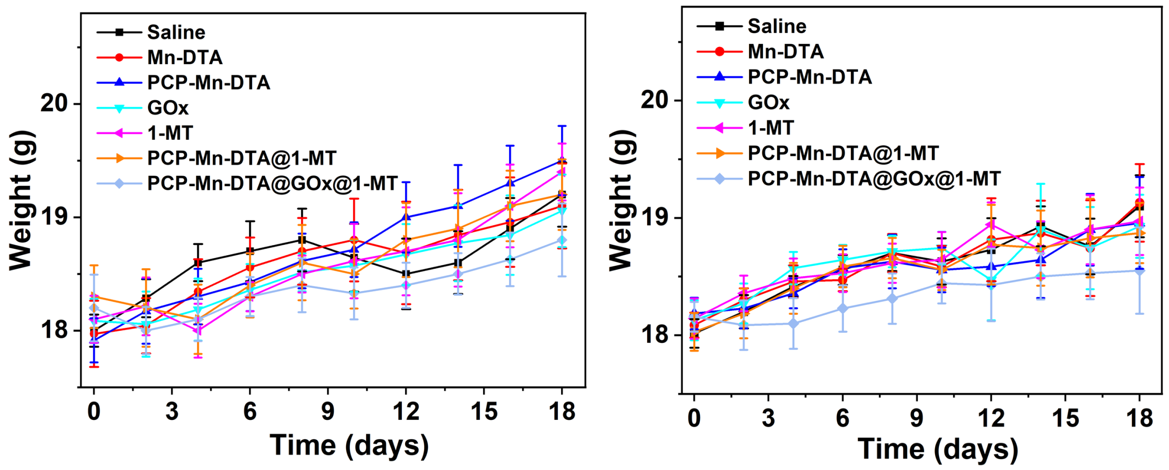

Supplementary Fig. 20. Weight change curves of mice. Weight change curves of mice after different treatments in (a) B16F10 and (b) 4T1 tumor models. Data represent mean ± SD (n = 6 biologically independent samples).

a

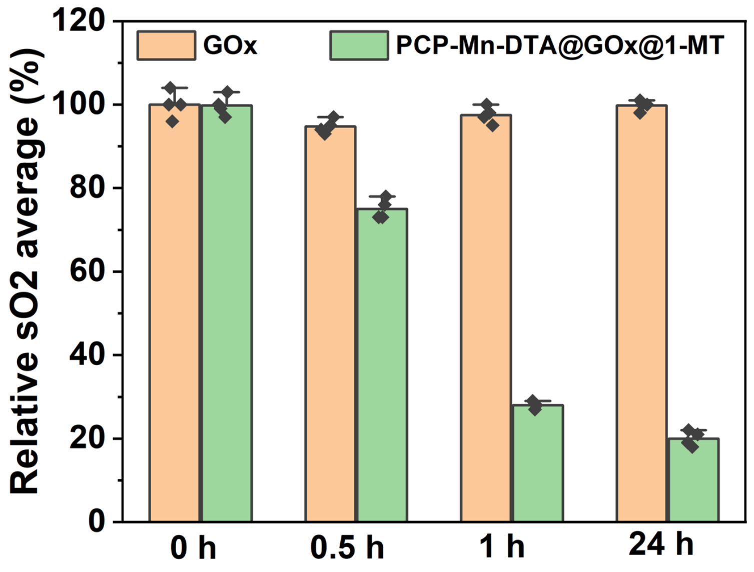

b

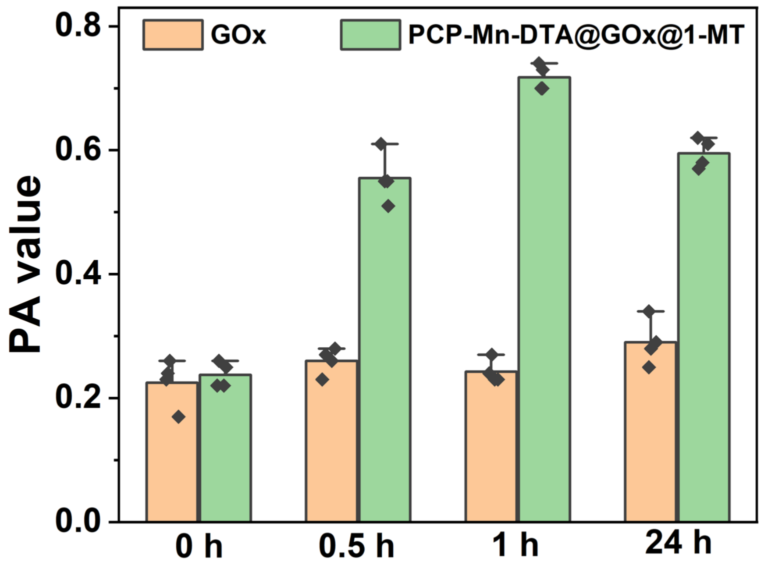

Supplementary Fig. 21. Quantification of sO_2 and photoacoustic (PA) values. (a) Quantification of sO_2 average total signals and (b) corresponding PA values of the tumor site after tumor-bearing mice were intratumorally injected with GOx or PCP-Mn-DTA@GOx@1-MT for various time intervals. Data represent mean ± SD (n = 4 biologically independent samples).

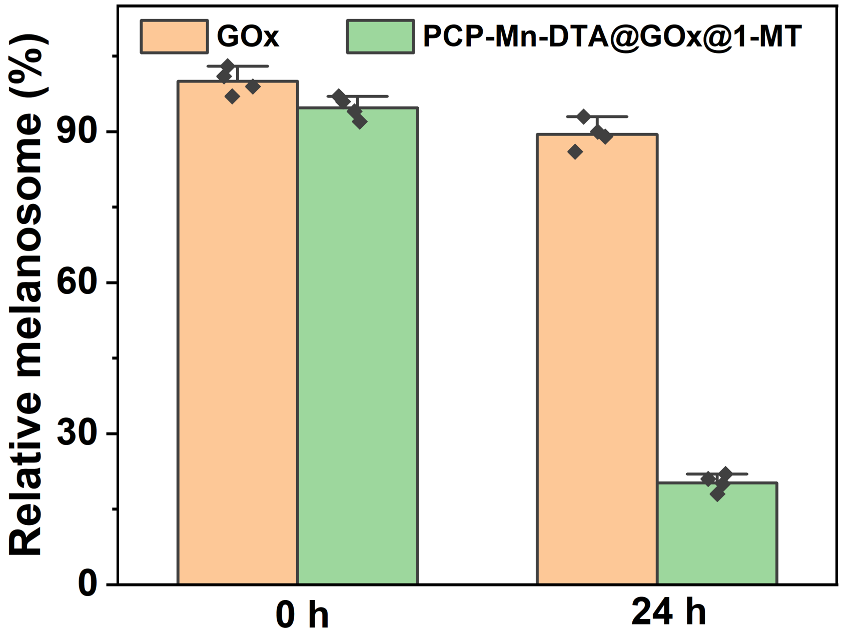

Supplementary Fig. 22. Quantitative melanosome signal analysis. Quantitative statistics of melanosome signals in tumor sites after tumor-bearing mice were intratumorally injected with GOx or PCP-Mn-DTA@GOx@1-MT for 24 h. Data represent mean ± SD (n = 4 biologically independent samples).

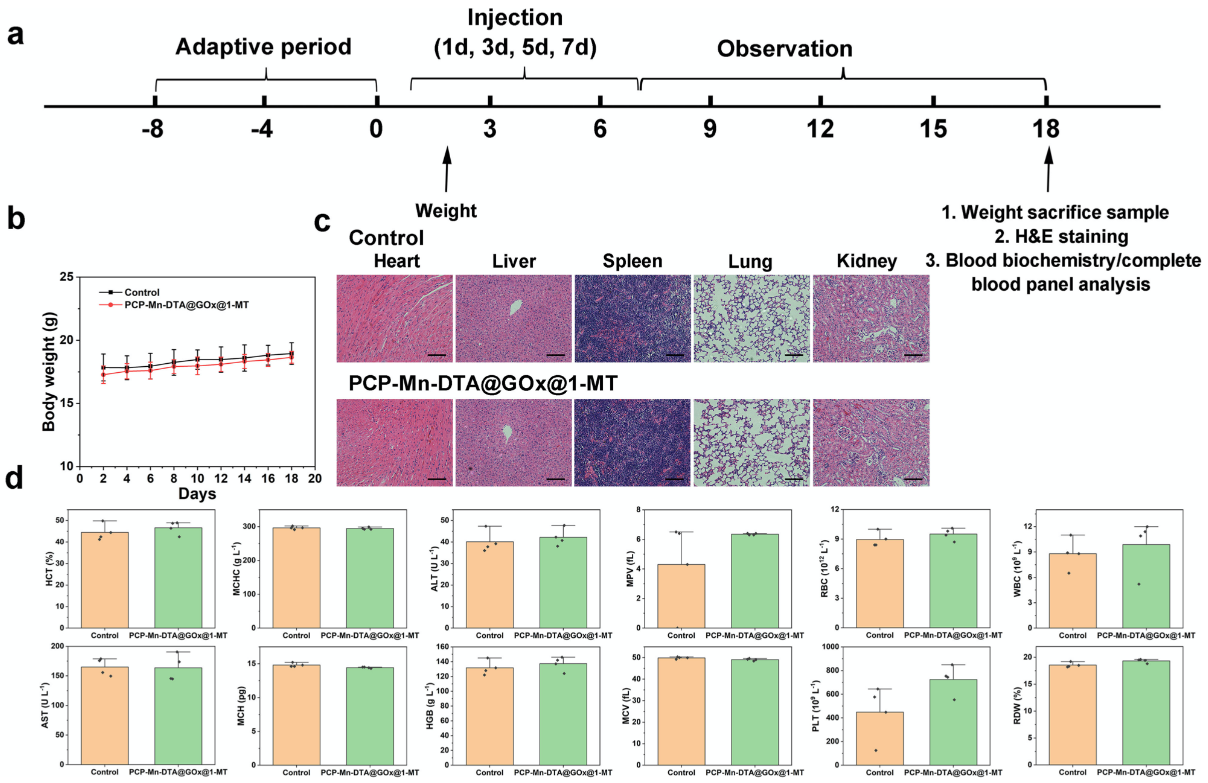

Supplementary Fig. 23. Toxicity studies. (a) Experimental design of repeated dose toxicity study of PCP-Mn-DTA@GOx@1-MT; (b) Body weights of mice after corresponding treatments; (c) Histological analysis of the major organs of untreated mice and mice treated with PCP-Mn-DTA@GOx@1-MT; (d) Blood biochemistry/complete blood panel analysis (hematocrit value (HCT), mean platelet volume (MPV), hemoglobin (HGB), platelets (PLT), mean corpuscular hemoglobin (MCH), red blood cells (RBC), mean corpuscular hemoglobin concentration (MCHC), red cell distribution width (RDW), mean corpuscular volume (MCV) and white blood cell (WBC)), liver function (aspartate transaminase (AST) and alanine aminotransferase (ALT)), and kidney function indexes (blood urea nitrogen (BUN) and creatinine (CREA)) of healthy mice and mice treated with PCP-Mn-DTA@GOx@1-MT for 18 days. Data represent mean ± SD (n = 4 biologically independent samples). Scale bar: 100 µm.

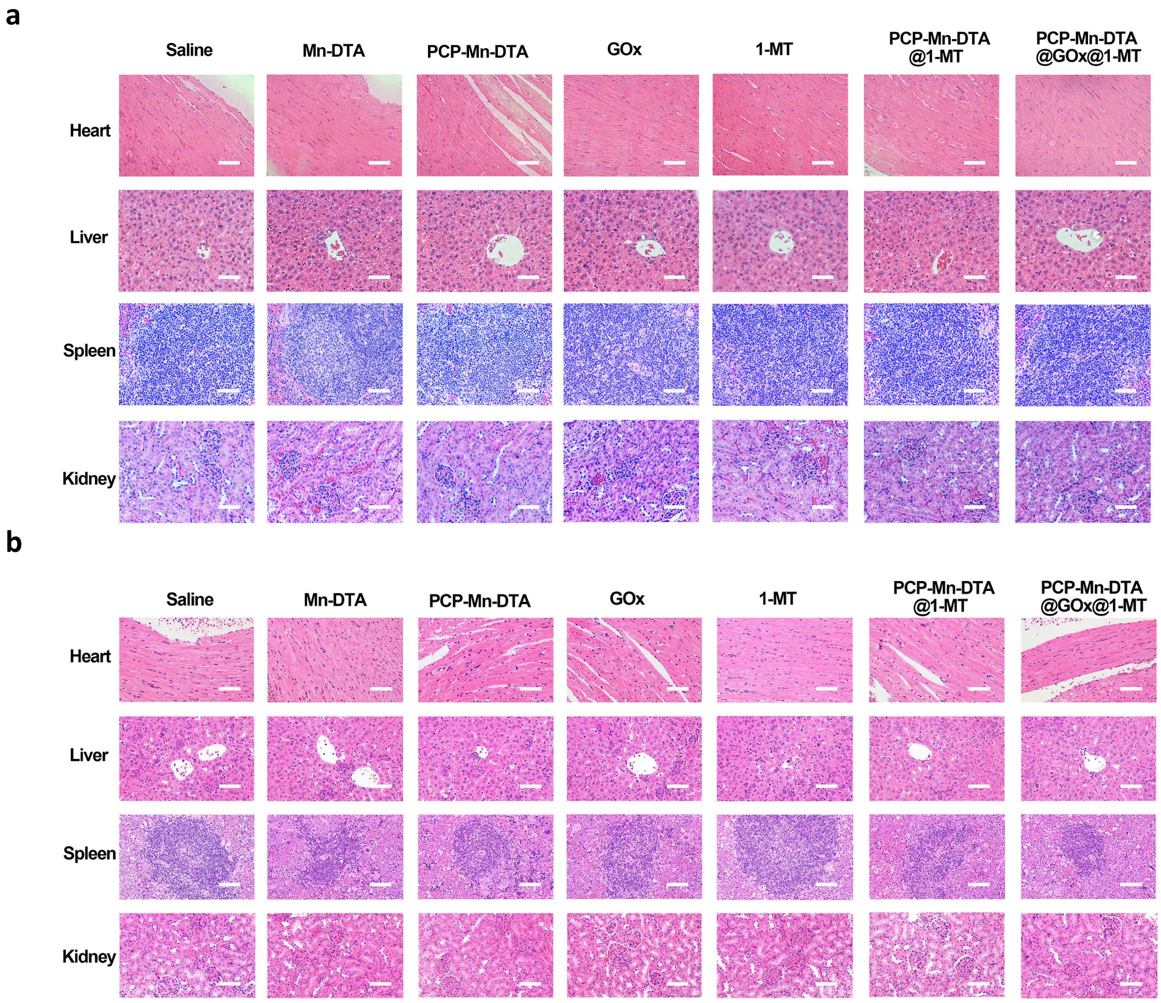

Supplementary Fig. 24. H&E staining analysis. H&E staining analysis of major tissues (heart, spleen, liver and kidney) of (a) B16F10 tumor-bearing C57BL/6 mice and (b) 4T1 tumor bearing Balb/c mice after treatments with various systems for 18 days. Scale bar: 100 µm.

a

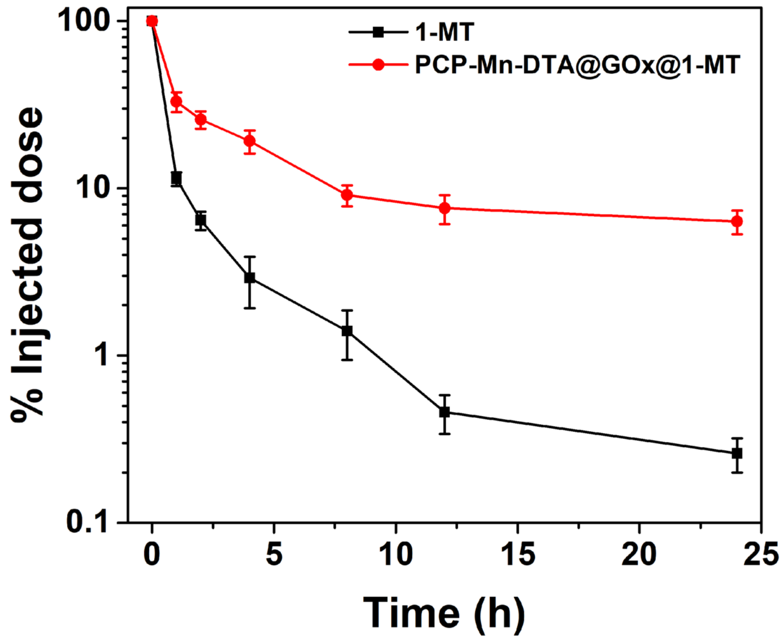

b

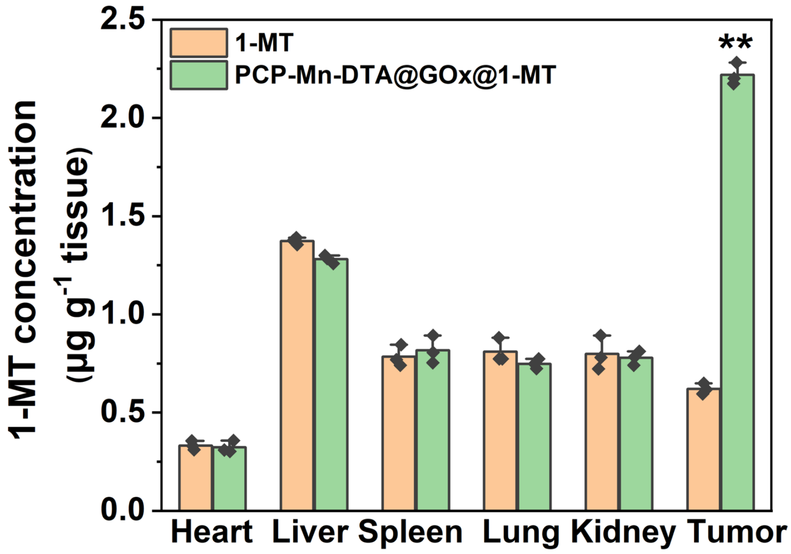

Supplementary Fig. 25. Pharmacokinetics and biodistribution. (a) Pharmacokinetics and (b) biodistribution of 1-MT and PCP-Mn-DTA@GOx@1-MT after intravenous injection into C57BL/6 mice at 1-MT dose of 3 mg/kg for 24 h. Data represent mean ± SD (n = 3 biologically independent samples). P-values were determined by unpaired Student's t-test (two-tailed), **p < 0.01.

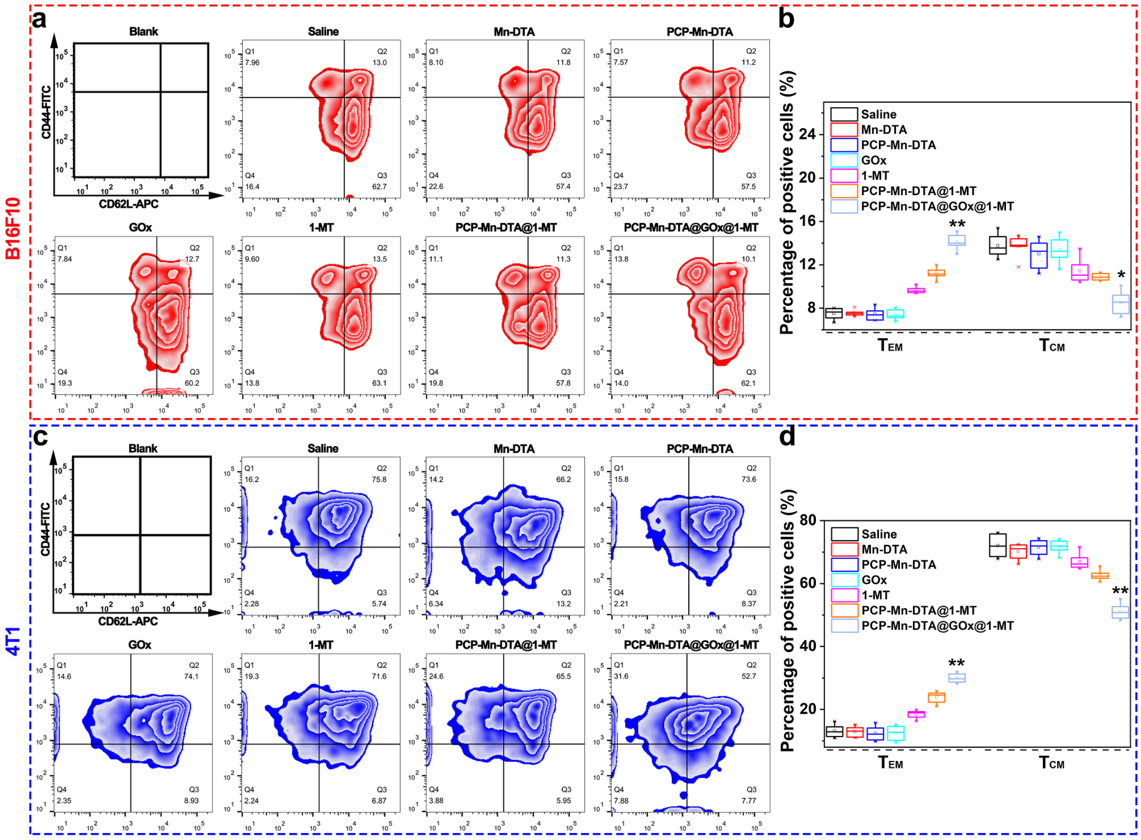

Supplementary Fig. 26. FCM studies. (a,c) FCM and (b,d) corresponding quantitative analysis on the frequency of T_{EM} and T_{CM} cells in the spleen at day 50 after tumor resection. Data represent mean ± SD (n = 6 biologically independent samples). P-values were determined by unpaired Student's t-test (two-tailed), * $p < 0.05$, ** $p < 0.01$. Box plots indicate median (middle line), 25th and 75th percentile (box), and 5th and 95th percentile (whiskers), as well as outliers (single points).

a

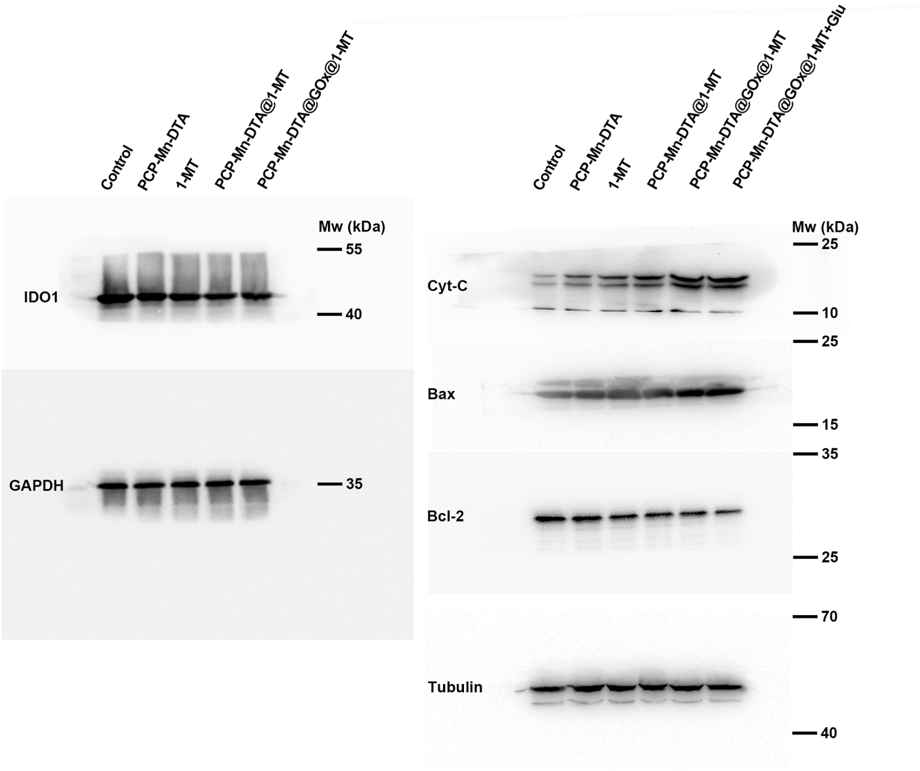

b

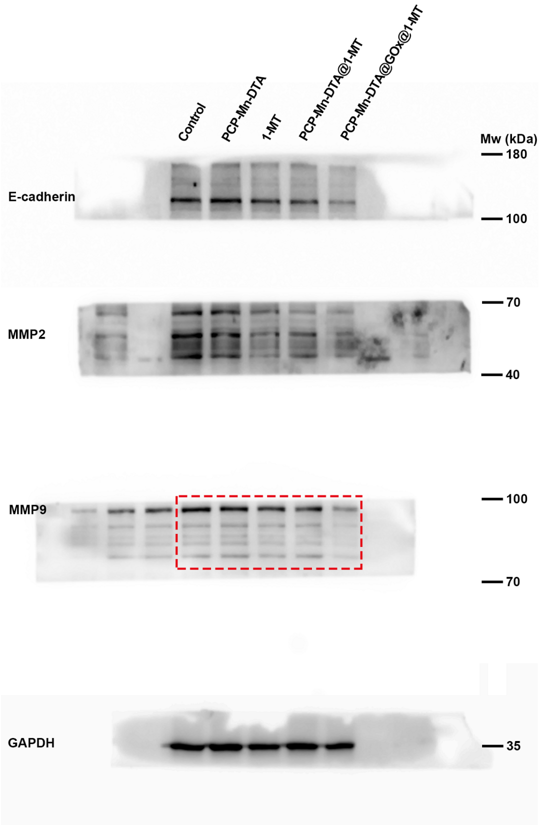

Supplementary Fig. 27. Uncropped scans of western blot with molecular weight markers. (a) Related results for Fig. 6a and Fig. 7d. (b) Related results for Supplementary Fig. 13a.

Supplementary Table 1. BET parameters of Mn-DTA nanoparticles.

Material	S_{BET} (m^2 g^{-1})	V_P (cm^3 g^{-1})	BJH (Å)
Mn-DTA	107.8	0.1934	86.1

Supplementary Table 2. Molecular weight data of PEG-CDM-PEI.

Polymer	Mn_{theory} (Da)	Mn_{NMR} (Da)	Mn_{GPC} (Da)	PDI
PEG-CDM-PEI	7153	6915	7200	1.29

Mn_{theory}: Theoretical molecular weight calculation based on monomer conversion.

Mn_{NMR}: Molecular weight based on ^1H NMR analysis.

Mn_{GPC}: Number-average absolute molecular weight determined by GPC equipped with refractive index and multi-angle light scattering detectors.

PDI: Polydispersity index determined by GPC analysis.
